# Endotoxin Translocation and Gut Barrier Dysfunction Are Related to Variceal Bleeding in Patients With Liver Cirrhosis

**DOI:** 10.3389/fmed.2022.836306

**Published:** 2022-03-03

**Authors:** Christos Triantos, Maria Kalafateli, Stelios F. Assimakopoulos, Katerina Karaivazoglou, Aikaterini Mantaka, Ioanna Aggeletopoulou, Panagiota I. Spantidea, Georgios Tsiaoussis, Maria Rodi, Hariklia Kranidioti, Dimitrios Goukos, Spilios Manolakopoulos, Charalambos Gogos, Dimitrios N. Samonakis, Georgios L. Daikos, Athanasia Mouzaki, Konstantinos Thomopoulos

**Affiliations:** ^1^Division of Gastroenterology, Department of Internal Medicine, University Hospital of Patras, Patras, Greece; ^2^Department of Internal Medicine, University Hospital of Patras, Patras, Greece; ^3^Department of Gastroenterology, University Hospital of Heraklion, Heraklion, Greece; ^4^Division of Hematology, Department of Internal Medicine, Medical School, University of Patras, Patras, Greece; ^5^2nd Department of Internal Medicine, Hippokration General Hospital of Athens, Athens, Greece; ^6^Department of Propedeutic Medicine, Laiko General Hospital, Athens, Greece

**Keywords:** cirrhosis, variceal bleeding, bacterial translocation, intestinal barrier, liver-gut axis

## Abstract

**Background:**

Bacterial infections are associated with the risk of variceal bleeding through complex pathophysiologic pathways.

**Objectives:**

The primary objective of the present case-control study was to investigate the role of bacterial translocation and intestinal barrier dysfunction in the pathogenesis of variceal bleeding. A secondary objective was to determine independent predictors of key outcomes in variceal bleeding, including bleeding-related mortality.

**Methods:**

Eighty-four (*n* = 84) consecutive patients participated in the study, 41 patients with acute variceal bleeding and 43 patients with stable cirrhosis, and were followed up for 6 weeks. Peripheral blood samples were collected at patient admission and before any therapeutic intervention.

**Results:**

Child-Pugh (CP) score (OR: 1.868; *p* = 0.044), IgM anti-endotoxin antibody levels (OR: 0.954; *p* = 0.016) and TGF-β levels (OR: 0.377; *p* = 0.026) were found to be significant predictors of variceal bleeding. Regression analysis revealed that albumin (OR: 0.0311; *p* = 0.023), CRP (OR: 3.234; *p* = 0.034) and FABP2 levels (OR:1.000, *p* = 0.040), CP score (OR: 2.504; *p* = 0.016), CP creatinine score (OR: 2.366; *p* = 0.008), end-stage liver disease model (MELD), Na (OR: 1.283; *p* = 0.033), portal vein thrombosis (OR: 0.075; *p* = 0.008), hepatocellular carcinoma (OR: 0.060; *p* = 0.003) and encephalopathy (OR: 0.179; *p* = 0.045) were significantly associated with 6-week mortality.

**Conclusions:**

Bacterial translocation and gut barrier impairment are directly related to the risk of variceal bleeding. Microbiota-modulating interventions and anti-endotoxin agents may be promising strategies to prevent variceal bleeding.

## Introduction

Acute variceal bleeding is a serious complication of liver cirrhosis associated with significant morbidity and mortality (1–3). Current treatment guidelines (4–6), which include early administration of vasoactive drugs, antibiotic prophylaxis, and band ligation, have resulted in increased survival (2).

The inclusion of prophylactic antibiotic treatment in current treatment guidelines is based on the notion that infection is a prognostic mortality factor (7, 8) and can also trigger variceal bleeding via a complex cascade of pathophysiologic events, including endotoxin release, endothelin activation, and portal hypertension (7, 9, 10). There is strong evidence that antibiotic use significantly reduces bacteremia and the incidence of spontaneous bacterial peritonitis, and that prophylactic antibiotic therapy prevents rebleeding and improves mortality (11–13). Moreover, bacterial infection has been shown to be strongly associated with variceal bleeding and failure to control bleeding (7). Nevertheless, the role of bacterial translocation in the pathogenesis of variceal bleeding has not been fully elucidated (14).

The aim of the present study was to determine whether bacterial translocation is more common in patients with acute variceal bleeding compared with stable cirrhotic patients. To this end, we examined indices of bacterial translocation and gut barrier integrity along with markers of systemic inflammatory response, including cytokine levels, in patients on admission to the hospital before any therapeutic manipulation that might increase the risk of infection and thus confound our results. Previous studies have shown indirect evidence of a contribution of bacterial translocation to variceal bleeding through activation of endogenous heparin-like activity (15, 16). However, there are no data directly linking bacterial translocation and endotoxin release to the risk of variceal bleeding. A secondary objective of the present study was to identify independent predictors of bleeding control failure, risk of rebleeding, and bleeding-related mortality.

## Materials and Methods

### Patients

The present case-control study was conducted in the Division of Gastroenterology, Department of Internal Medicine, Patras University General Hospital (PUH), Patras, Greece. All study participants or their legal guardians provided written informed consent before participating in the study. The study protocol was approved by the Scientific Review Board and the Ethics Committee of PUH as part of a general application to collect biological samples from patients attending the hepatology clinics to study factors involved in the pathogenesis of liver cirrhosis. PUH adheres to the 1975 Declaration of Helsinki on Ethical Principles for Medical Research Involving Human Subjects.

We studied 41 consecutive patients with cirrhosis and variceal bleeding and 43 stable consecutive patients with cirrhosis who visited the hepatology outpatient clinics from February 2016 to February 2018. Patients were followed for 6 weeks. Patients with variceal bleeding were treated according to the guidelines at that time (5, 6). The presence of infection was investigated with blood and urine cultures, paracentesis and culture of ascites, and chest radiography. The presence of active infection at any site was an exclusion criterion because we wanted to focus on the role of bacterial translocation in variceal bleeding.

### Diagnosis

Liver cirrhosis was diagnosed on the basis of histologic findings, clinical evaluation, laboratory data, or imaging findings. Variceal bleeding was diagnosed based on hematemesis or melena with either bleeding varix (active bleeding or clot adherent to the varix or variceal ulceration) or, if there was no other source of bleeding, during upper gastrointestinal endoscopy. Severe bleeding was defined as arterial hypotension (systolic blood pressure < 100 mm Hg) or/and hemoglobin < 8 g/dl on admission. Failure of bleeding control was defined as recurrence of bleeding during the 5 days of somatostatin infusion. Rebleeding was defined as the recurrence of bleeding between days 5 and 42 after the initial bleeding. Bleeding-related mortality was defined as death within 6 weeks of the bleeding episode (17).

### Markers of Systemic Inflammation, Bacterial Translocation, and Gut Barrier Integrity

Peripheral blood samples (3-4 ml) were collected at patient admission before therapeutic intervention. Blood samples were centrifuged, and sera were collected and stored aliquoted at −80°C until further use.

Systemic inflammation was assessed by measuring C-reactive protein (CRP), erythrocyte sedimentation rate (ESR), nitric oxide (NO), soluble CD14, and pro- and anti-inflammatory cytokine levels (IL-1β, IL-6, IL-8, IL-12, TNF-α, IL-10, TGF-β). The presence of bacterial translocation was estimated by measuring serum lipopolysaccharide (LPS), anti-endotoxin immunoglobulin M (IgM) and immunoglobulin G (IgG) antibodies, lipopolysaccharide-binding protein (LBP), and bacterial DNA. Gut barrier integrity was assessed by measuring fatty acid binding protein 2 (FABP2) in serum.

### Measurement of Serum Levels of Cytokines, LBP, Endotoxin, and NO

Serum levels of cytokines IL-1β, IL-6, IL-8, IL-10, IL-12p70, and TNF-α were determined by a cytometric bead array (CBA) method using the Human Inflammatory Cytokine CBA kit (BD Bioscience, San Diego, USA) as described (18). Measurements were performed using a BD FACS Array Bioanalyzer as described (18). Raw data were analyzed using Flow Jo V7.5 software (Tree Star Inc., Ashland, OR, USA). Serum TGF-β and LBP concentrations were determined using a multispecies TGF-β ELISA kit (Invitrogen Corporation, CA, USA) and a human LBP ELISA kit (SunRed Biological Technology, Shanghai) respectively, as described in (18). NO levels were measured using a nitric oxide quantification kit (Active Motif, Belgium), as described in (18). Endotoxin levels were measured using the Limulus amebocyte lysate chromogenic endpoint assay (Hycult Biotech, The Netherlands), as described in (18). Data analysis was performed using Curve Expert 1.4 software. The detection limits of the methods used were: IL-1β = 7.2 pg/ml, IL-8 = 3.6 pg/ml, IL-10 = 3.3 pg/ml, IL-6 = 2.5 pg/ml, IL12p70 = 1.9 pg/ml, TNF-α = 3.7 pg/ml, TGF-β = 15.6 pg/ml, LBP = 0.135 μg/ml, NO < 1 μM, endotoxin = 0.04 EU/ml.

### Detection of Bacterial DNA in Serum

Bacterial DNA was detected by PCR using a pair of universal primers (F: 5′-AGAGTTTGATCATGGCTCAG-3′, R: 5′-ACCGCGACTGCTGCTGGCAC-3′) as described in (18, 19).

### Statistical Analysis

Numerical data were expressed as medians and interquartile ranges (IQR) and categorical data as counts and percentages. All variables were tested for normal distribution using the Kolmogorov-Smirnov test. According to this analysis, the values of variables did not follow a normal distribution. For this reason, comparisons between groups were made using the Mann-Whitney test, which is a non-parametric test. Differences between groups in demographic, clinical, and laboratory parameters were assessed with the χ^2^ test for categorical variables. A number of clinically relevant variables that differed significantly between bleeders and stable cirrhotics entered multivariate stepwise logistic regression analysis to determine which parameters better predicted bleeding risk. Collinearity among the independent variables was tested using variance inflation factors and tolerances for each variable. Given the presence of statistical collinearity, individual variables required for calculation of the end-stage liver disease model (MELD) or Child-Pugh (CP) score were excluded from multivariate analysis. *P* < 0.05 was considered to indicate a statistically significant difference. The SPSS statistical package (version 19.0 for Windows; SPSS Inc., Chicago, Illinois, USA) was used. Graphical representation was performed using GraphPad Prism v. 8.3.1 software.

## Results

Eighty-four consecutive patients participated in the study, 41 patients with acute variceal bleeding and 43 patients with stable cirrhosis. Three patients with variceal bleeding had evidence of active infection, 2 had urinary tract infection, and 1 had a positive blood culture; these patients were excluded from further analysis. Six patients (15.8%) with variceal bleeding had active bleeding at endoscopy. As expected, patients with variceal bleeding were more likely to have been treated with beta-blockers compared to the control group (*p* = 0.021). Similarly, patients with variceal bleeding were more likely to have higher Child-Pugh (*p* = 0.042) and MELD scores (*p* < 0.001).

**Figure 1:**
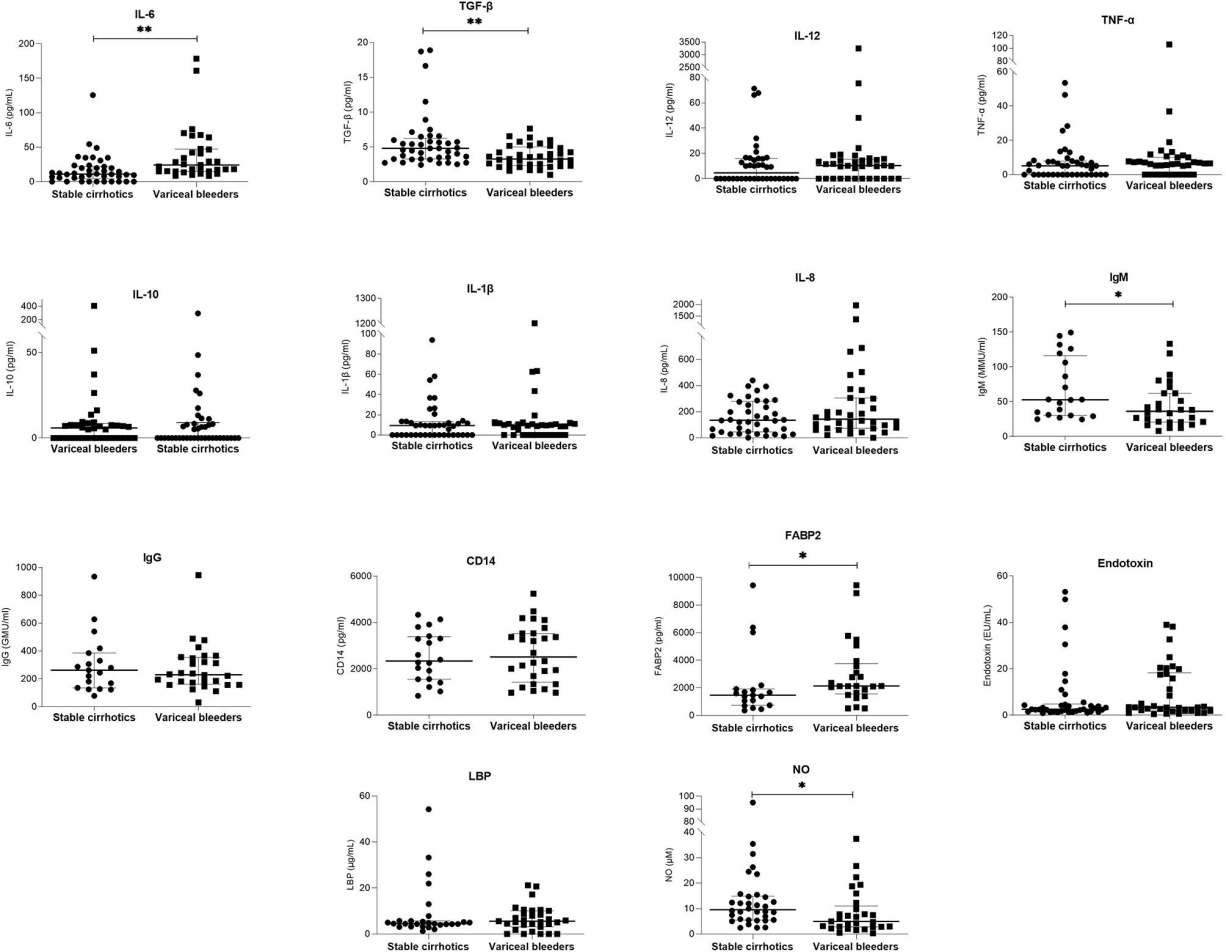
Comparison of serum laboratory parameters between patients with stable liver cirrhosis and patients presenting with variceal bleeding. Bars and plots represent the median and interquartile range, respectively. Asterisks indicate statistical significance (*p < 0.05; **p < 0.01).

Table 1 shows the demographic and clinical parameters of stable cirrhotic patients compared to patients with variceal bleeding. Patients with variceal bleeding had significantly lower hemoglobin and albumin levels and significantly higher white blood cell counts compared to controls. In addition, variceal bleeders had significantly lower IgM anti-endotoxin antibody, NO, and TGF-β levels and higher FABP2 and IL-6 levels (Figure 1 and Supplementary Table 1). Bacterial DNA was not detected in any of the serum samples.

**Table 1:** Demographic, clinical, and laboratory data of study subjects.

	**All study subjects**	**Variceal bleeders**	**Stable cirrhotics**	***P*-value**
N	84	38	43	
Gender, male (%)	62 (76.5)	32 (84.2)	30 (69.8)	0.126
Smoking, yes (%)	29 (43.9)	17 (58.6)	12 (32.4)	0.052
Past variceal bleeding, yes (%)	28 (43.1)	18 (50.0)	10 (34.5)	0.209
Use of beta-blockers, yes (%)	38 (46.9)	23 (60.5)	15 (34.9)	**0.021**
HCC, yes (%)	12 (14.8)	8 (21.1)	4 (9.3)	0.137
Portal vein thrombosis, yes (%)	9 (11.3)	6 (16.2)	3 (7.0)	0.192
Ascites, yes (%)	34 (42.0)	20 (52.6)	14 (32.6)	0.068
Encephalopathy, yes (%)	12 (14.8)	9 (23.7)	3 (7.0)	**0.035**
	**Median (IQR)**			
Age	60 (52, 65.5)	57.5 (47.5, 66.5)	61 (54, 65)	0.656
Child-Pugh score	7.0 (6, 9)	8 (7, 9)	6 (5, 8.8)	**0.042**
MELD score	12 (10, 16)	14.5 (11, 16.25)	10 (8, 13)	**<0.001**
MELDNa score	15 (12, 18)	16 (14, 19)	13 (9, 15.5)	**0.001**
Hemoglobin (g/dl)	10.8 (9.2, 13.5)	9.2 (7.9, 10.0)	12.8 (10.9, 14.7)	**<0.001**
WBC (cells/μl)	6355 (4602.3, 9522.5)	9160 (6730, 13350)	4830 (4200, 6252.5)	**<0.001**
Albumin (g/dl)	3.4 (2.8, 4.0)	3 (2.6, 3.4)	3.7 (3.2, 4.2)	**<0.001**
ESR (mm/hour)	40.0 (35-60)	40.0 (30.5-62.5)	45 (40, 50)	0.798
CRP (mg/L)	0.8 (0.4-2.0)	1.3 (0.4-2.3)	0.4 (0.1, 0.8)	0.231
Bacterial DNA	ND	ND	ND	ND

*HCC, hepatocellular carcinoma; IQR, interquartile range; MELD, model of end-stage liver disease; WBC, white blood cells; ESR, erythrocyte sedimentation rate; CRP, C-reactive protein; ND, not detected. The bold values correspond to statistical significant differences between the examined variables*.

Multivariate stepwise regression was then calculated to predict variceal bleeding risk based on Child-Pugh score, beta-blocker treatment, IgM anti-endotoxin antibodies, serum levels of TGF-β and IL-6. The results (Table 2) show that Child-Pugh score, IgM anti-endotoxin antibodies, and TGF-β levels remained as significant predictors of variceal bleeding and explained 48.9% of the variance in variceal bleeding risk.

**Table 2:** Independent predictors of variceal bleeding.

	**Univariate analyses**	**Multivariate stepwise regression analysis**		
	***P*-value**	**OR**	**95% CI for exp (B)**	***P*-value**
IgM (GMU/ml)	0.049	0.954	0.917-0.991	**0.016**
TGF-β (pg/ml)	0.009	0.377	0.159-0.891	**0.026**
Child-Pugh score	0.126	1.868	1.016-3.433	**0.044**
IL-6 (pg/ml)	0.024	-	-	0.204
Beta-blockers treatment	0.023	-	-	0.320
FABP2 (pg/ml)	0.250	-	-	0.511
Regression statistics		X^2^ = 22.895, df = 4, *p* < 0.001 Nagelkerke R^2^ = 0.583		

*OR, odds ratio; CI, confidence interval; FABP2, fatty acid-binding protein 2. The bold values correspond to statistical significant differences between the examined variables*.

### Failure to Control Bleeding and Rebleeding

**Figure 2:**
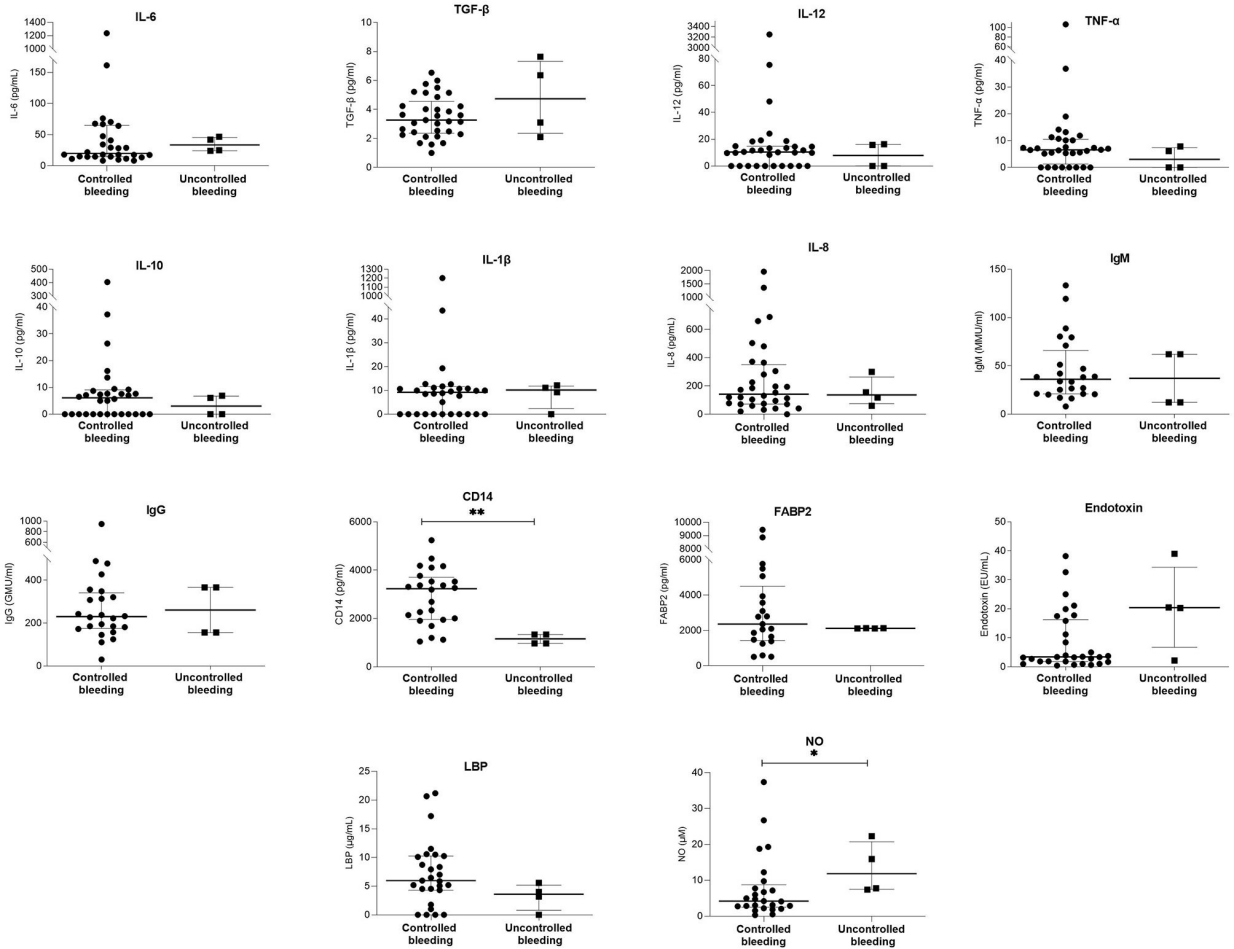
Comparison of serum laboratory parameters between patients with controlled bleeding and patients with uncontrolled bleeding. Bars and plots represent median and interquartile range, respectively. Asterisks indicate statistical significance (*p < 0.05; **p < 0.01).

Bleeding control was unsuccessful in 4 (10.5%) patients. After initial bleeding control, rebleeding occurred in 3 (7.9%) patients between day 5 and day 42. Table 3; Figure 2 and Supplementary Table 2 show the demographic, clinical, and laboratory parameters of patients whose bleeding could not be initially controlled compared with those whose bleeding was successfully controlled. Table 4 shows the demographic, clinical, and laboratory parameters of the patients in whom rebleeding occurred compared with the patients in whom rebleeding did not occur. Multivariate regression analysis to determine independent predictors of bleeding control failure and risk of rebleeding was not performed because of the small sample size.

**Table 3:** Demographic, clinical and laboratory parameters of patients with uncontrolled bleeding vs. patients with controlled bleeding.

**Variceal bleeders**	**Failure to control bleeding**	**Controlled bleeding**	***P*-value**
*N*	4	34	
Gender, male (%)	4 (100)	28 (82.4)	0.360
Smoking, yes (%)	2 (50)	15 (60.0)	0.706
Past variceal bleeding, yes (%)	0 (0)	18 (54.5)	0.070
Use of beta-blockers, yes (%)	3 (75)	20 (58.8)	0.482
HCC, yes (%)	2 (50)	6 (17.6)	0.133
Portal vein thrombosis, yes (%)	1 (33.3)	5 (14.7)	0.401
Ascites, yes (%)	3 (75)	17 (50.0)	0.344
Encephalopathy, yes (%)	2 (50)	7 (20.6)	0.191
	**Median (IQR)**		
Age	61.5 (53.3, 70.5)	57.5 (44.5, 66.5)	0.536
Child-Pugh score	9 (8.3, 9.8)	8 (6.8, 9)	0.100
Child-Pugh creatinine score	9 (8.3, 9.8)	8 (6.5, 9)	0.124
MELD	16 (12, 17)	14 (11, 16)	0.444
MELDNa	19 (17, 21.8)	16 (14, 18.3)	0.069
Hemoglobulin (g/dl)	10.0 (8.9, 11.9)	9.1 (7.8, 10.0)	0.208
WBC (cells/μl)	14800 (9995, 18450)	8630 (6470, 13000)	0.058
Albumin (g/dl)	2.6 (2.3, 2.9)	3.1 (2.7, 3.5)	**0.048**

*HCC, hepatocellular carcinoma; IQR, interquartile range; MELD, model of end-stage liver disease; WBC, white blood cells. The bold values correspond to statistical significant differences between the examined variables*.

**Table 4:** Demographic, clinical and laboratory parameters of rebleeders vs. non-rebleeders.

	**Rebleeders**	**Non-rebleeders**	***P*-value**
*N*	3	35	
Gender, male (%)	3 (100)	29 (82.9)	0.435
Smoking, yes (%)	2 (66.7)	15 (57.7)	0.765
Past variceal bleeding, yes (%)	2 (66.7)	16 (48.5)	0.546
Use of beta-blockers, yes (%)	2 (66.7)	21 (60.0)	0.821
HCC, yes (%)	1 (33.3)	7 (20.0)	0.587
Portal vein thrombosis, yes (%)	1 (33.3)	5 (14.7)	0.401
Ascites, yes (%)	1 (33.3)	19 (54.3)	0.485
Encephalopathy, yes (%)	0 (0)	9 (25.7)	0.315
	**Median**		
Age	36.35	58	0.588
Child-Pugh score	9	8	0.563
Child-Pugh creatinine score	9	8	0.337
MELD	13	15	0.935
MELDNa	17	16.0	0.913
Hemoglobulin (g/dl)	8	9.3	0.616
WBC (cells/μl)	9260	8930	0.953
Albumin (g/dl)	2.4	3.1	0.076
ESR (mm/h)	41.5	40.0	0.921
CRP (mg/L)	4.74	0.97	0.146
IgM (MMU/ml)	36.35	36.0	0.923
IgG (GMU/ml)	193.75	229.8	0.501
FABP2 (pg/ml)	5463.55	2247.2	0.465
CD14 (pg/ml)	3429.95	2768.2	0.386
TGF-β (pg/ml)	5.85	3.2	0.065
IL-1β (pg/ml)	600.82	9.4	0.699
IL-6 (pg/ml)	624.23	23.1	0.730
IL-8 (pg/ml)	420.97	125.9	0.269
IL-12 (pg/ml)	13.49	10.4	0.622
IL-10 (pg/ml)	202.68	5.9	0.616
TNFα (pg/ml)	8.76	6.4	0.530
Endotoxin (EU/ml)	5.15	3.4	0.763
LBP (μg/ml)	8.55	5.4	0.359
NO (μM)	5.96	5.0	0.796

*HCC, hepatocellular carcinoma; MELD, model of end-stage liver disease; WBC, white blood cells; ECR, erythrocyte sedimentation rate; CRP, C-reactive protein; FABP2, fatty acid-binding protein 2; LBP, lipoprotein binding protein; NO, nitrogen oxide*.

### 6-Week Mortality

**Figure 3:**
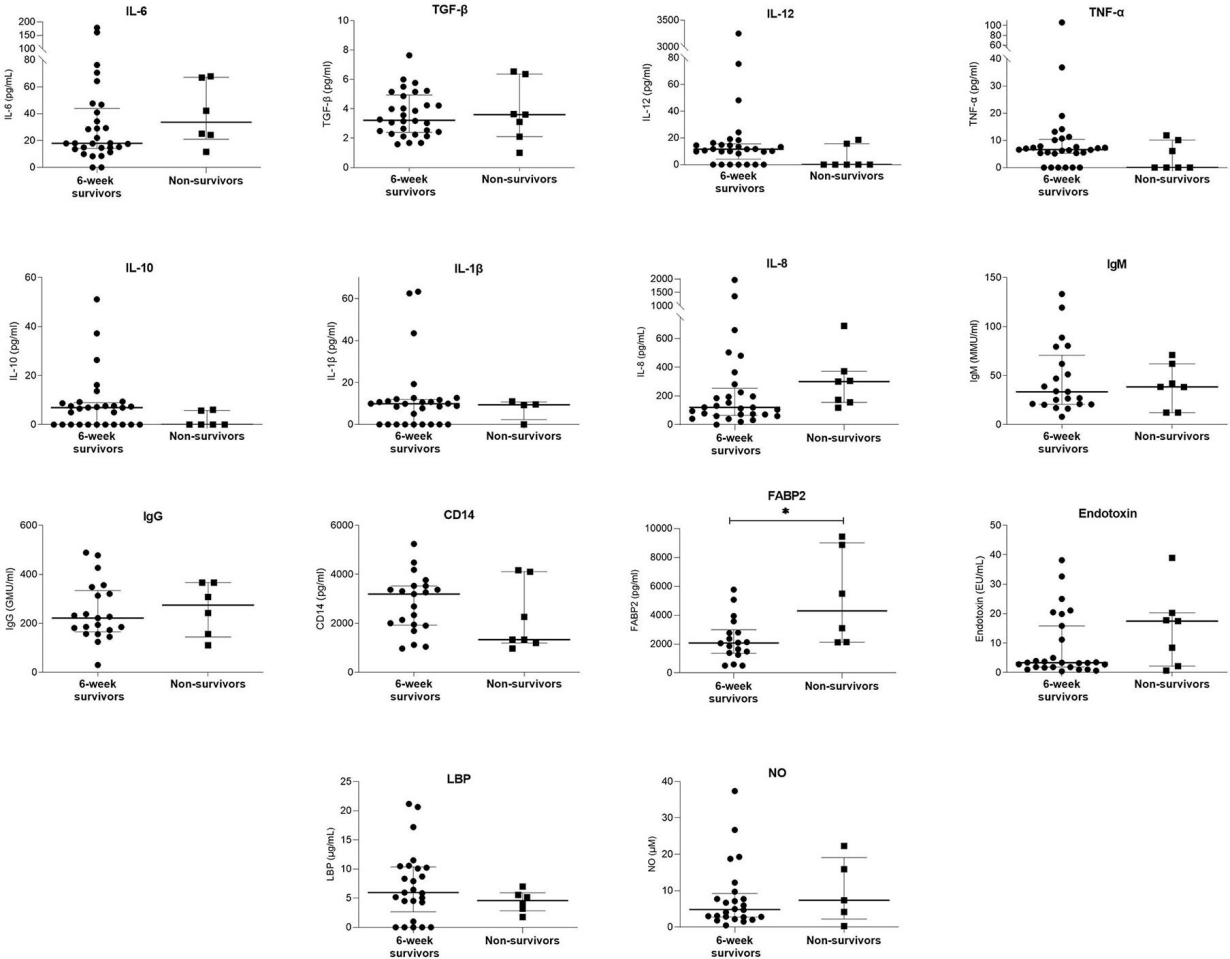
Comparison of serum laboratory parameters between 6-week survivors and non-survivors. Bars and plots represent median and interquartile range, respectively. Asterisks indicate statistical significance (*p < 0.05).

Eight (21.1%) of variceal bleeders died within 6 weeks of the bleeding episode. Table 5; Figure 3 and Supplementary Table 3 show a comparison of demographic, clinical, and laboratory data between the 6-week survivors and non-survivors in the variceal bleeding group. Binary logistic regression analysis was performed to determine the variables that significantly predicted death within 6 weeks in the variceal bleeders group. After univariate analysis, the levels of albumin, CRP, and FABP2, Child-Pugh score, Child-Pugh creatinine score, MELDNa, presence of portal vein thrombosis, and hepatocellular carcinoma were significantly associated with 6-week mortality. Table 6 shows the coefficients and odds ratios for each variable included in the univariate regression analysis for 6-week mortality.

**Table 5:** Demographic, clinical and laboratory parameters in 6-week survivors vs. non-survivors.

**Variceal bleeders**		**6-week survivors**	**Non-survivors**	***P*-value**
N	30	8		
Cause of death, yes (%)	Bleeding	NA	4 (50)	NA
	Sepsis		1 (12.5)	
	HCC		3 (37.5)	
	Renal failure		4 (50)	
	Liver failure		4 (50)	
Gender, male (%)		24 (80.0)	8 (100)	0.168
Smoking, yes (%)		14 (63.9)	3 (42.9)	0.331
Past variceal bleeding, yes (%)		17 (58.6)	1 (14.3)	**0.035**
Use of beta-blockers, yes (%)		16 (53.3)	7 (87.5)	0.079
HCC, yes (%)		3 (10.0)	5 (62.5)	**0.001**
Portal vein thrombosis, yes (%)		2 (6.7)	4 (57.1)	**0.001**
Ascites, yes (%)		16 (53.3)	4 (50)	0.867
Encephalopathy, yes (%)		5 (16.7)	4 (50)	**0.049**
	**Median (IQR)**			
Age		54.5 (39.8, 65.3)	66 (59, 74.3)	**0.023**
Child-Pugh score		7.5 (6.0, 8.3)	9 (9, 10)	**0.002**
Child-Pugh creatinine score		7 (6.0, 8)	9.5 (9, 11.75)	**0.001**
MELD		14 (11, 16)	17 (13.5, 18.5)	**0.039**
MELDNa		16 (13.8, 17)	20 (17.25, 21.75)	**0.002**
Hemoglobulin (g/dl)		8.9 (7.8, 9.9)	10 (8.8, 12.4)	0.079
WBC (cells/μl)		8500 (6300, 11410)	13175 (9185, 16525)	**0.036**
Albumin (g/dl)		3.2 (2.8, 3.5)	2.4 (2.2, 2.8)	**0.005**
ESR (mm/h)		45 (36.5, 71.3)	28 (21.5, 39.8)	0.176
CRP (mg/L)		0.6 (0.4, 1.5)	3.62 (1.61, 5.66)	**0.021**

*HCC, hepatocellular carcinoma; NA, not applicable; IQR, interquartile range; MELD, model of end-stage liver disease; WBC, white blood cells; ECR, erythrocyte sedimentation rate; CRP, C-reactive protein. The bold values correspond to statistical significant differences between the examined variables*.

**Table 6:** Independent predictors of 6-week mortality risk.

		**Univariate logistic regression analysis**		
	**Coefficient**	**OR**	**95% CI for exp (B)**	***P*-value**
Age (years)	0.078	1.081	1.004-1.165	**0.038**
WBC (cells/μl)	0.000	1.000	1.000-1.000	0.065
Albumin (g/dl)	−3.562	0.028	0.002-0.527	**0.017**
Child-Pugh score	1.204	3.334	1.304-8.522	**0.012**
Child-Pugh creatinine score	1.012	2.751	1.301-5.817	**0.008**
MELD score	0.232	1.262	0.973-1.635	0.079
MELDNa score	0.416	1.515	1.106-2.077	**0.010**
FABP2 (pg/ml)	0.001	1.000	1.000-1.001	**0.040**
CRP (mg/L)	1.128	3.090	1.061-8.999	**0.039**
Past variceal bleeding	−2.140	0.118	0.012-1.108	0.061
Beta-blockers treatment	1.812	6.125	0.669-56.095	0.109
HCC	2.708	15.000	2.328-96.666	**0.004**
Portal vein thrombosis	2.927	18.667	2.348-148.426	**0.006**
Encephalopathy	1.609	5.0	0.926-26.990	0.061

*OR, odds ratio; CI, confidence interval; WBC, white blood cells; MELD, model of end-stage liver disease; FABP2, fatty acid-binding protein 2; CRP, C-reactive protein; HCC, hepatocellular carcinoma. The bold values correspond to statistical significant differences between the examined variables*.

## Discussion

In the present study, the vast majority of variceal bleeders had no evidence of overt infection on hospital admission before diagnostic or therapeutic intervention. However, we observed significantly lower levels of IgM anti-endotoxin antibodies and TGF-β in bleeders compared to non-bleeders. The absence of a difference in anti-endotoxin IgG antibodies between variceal bleeders and non-bleeders suggests a chronic exposure of both cirrhotic groups to low-grade endotoxemia, whereas the significantly lower anti-endotoxin IgM levels in variceal bleeders are consistent with a pulse of recent endotoxin release leading to antibody consumption (20, 21). This is the first study to provide direct evidence that bacterial translocation is associated with variceal bleeding, possibly suggesting that the latter is a critical precursor to increased bleeding risk (22).

Bacterial translocation is commonly found in cirrhotics (23) and has been associated with hemodynamic changes and portal hypertension (9, 10, 24). The passage of microbes and their products from the intestinal lumen into the mesenteric lymph nodes and systemic circulation stimulates a cascade of intrahepatic immune signals that promote fibrosis and changes in intrahepatic vascular tone, leading to an exacerbation of portal hypertension (24). In addition, the presence of bacterial infection in cirrhotic patients has been associated with increased endogenous heparin-like activity, which inhibits platelet aggregation and leads to a higher risk of bleeding (16).

Goulis et al. (25) formulated the hypothesis that bacterial infection induced by bacterial translocation could be the event that triggers variceal bleeding through a combination of physiological phenomena, including impaired coagulation, increased portal pressure, and failure of liver function. This hypothesis is primarily based on a previous study by the same group, which showed that the presence of bacterial infection is an independent predictor of failure to control bleeding in patients with variceal bleeding (7). Previous studies have also shown that bacterial infections affect 20-60% of variceal bleeders (7, 26, 27) and that the presence of bacterial infection independently predicts the risk of rebleeding and mortality (7, 26, 28, 29). However, the methodological design of these studies did not allow for differentiation between infections that were already present at the time of bleeding and infections that occurred as a consequence of bleeding or due to the invasive nature of endoscopic procedures (14). In addition, several studies have reported that the prophylactic use of antibiotics reduces infection rates, the risk of rebleeding, and all-cause mortality (29–31). A recent meta-analysis found that the relevant studies had methodological weaknesses and a high risk of bias, suggesting that further research on this topic is needed (12). In addition, Goulis' hypothesis focused exclusively on the role of infection and did not consider that the phenomena of gut barrier dysfunction and bacterial translocation do not always lead to infection but may still be related to the pathophysiology of variceal bleeding.

In the present study, emphasis was placed on measuring indices of bacterial translocation as close as possible to the bleeding episode and before any intervention, and patients with active infection at any site or bacteremia were excluded to ensure that the endotoxin detected originated from the intestinal lumen. Our results strongly suggest a close pathophysiological relationship between the translocation of bacteria and endotoxins from the intestine and variceal bleeding. Although our results do not provide evidence of causality, the finding that variceal bleeders had decreased levels of IgM anti-endotoxin antibodies in the absence of bacterial DNA at the onset of bleeding suggests that endotoxin translocation may be the initiating event in the pathophysiology of variceal bleeding. Previous studies have shown that increased gut permeability precedes the detection of bacterial DNA in the sera of cirrhotic patients (22, 32). In addition, detection of endotoxin may be a more reliable index of bacterial translocation than bacterial DNA; furthermore, the presence of bacterial byproducts in patients' sera likely represents a later step in the timeline of variceal bleeding pathophysiology (19). Bacterial translocation is a transient phenomenon that does not always lead to variceal bleeding. Our analysis showed that bleeders and the cirrhotic patients had similar levels of IgG anti-endotoxin antibodies suggestive of previous episodes of bacterial translocation. This confirms that cirrhotic patients often experience repeated episodes of bacterial translocation or chronic low-grade endotoxemia. The fact that variceal bleeders exhibited stronger evidence of recent bacterial translocation strengthens the possibility that this is the trigger of the bleeding.

**Figure 4:**
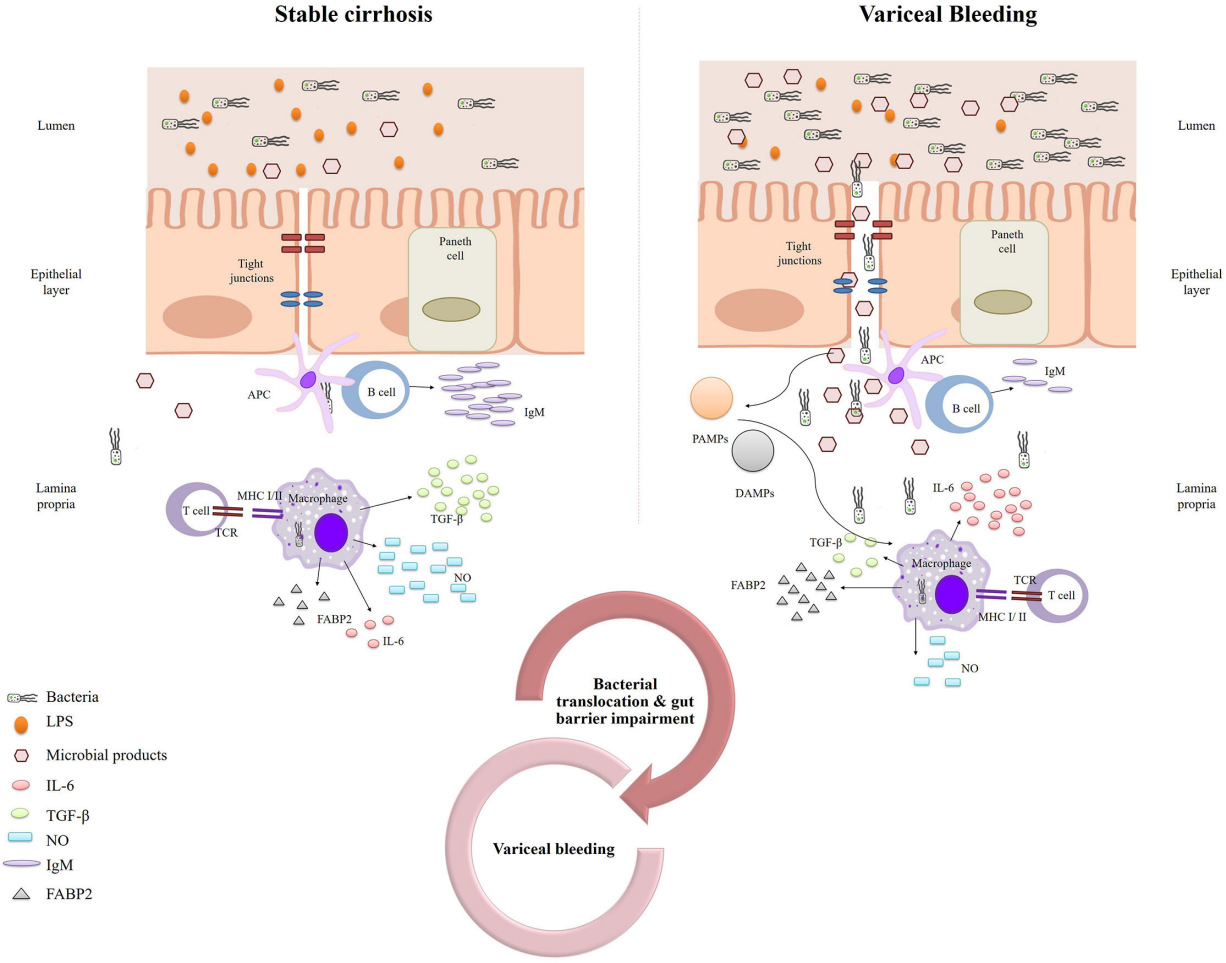
Schematic representation of our proposed mechanism for the contribution of bacterial translocation and increased gut permeability to variceal bleeding risk.

The central role of bacterial and endotoxin translocation (due to a disrupted intestinal barrier) in variceal bleeding is further supported by our finding of increased FABP2 levels in variceal bleeders. Moreover, according to our analysis, FABP levels proved to be a significant predictor of 6-week mortality in variceal bleeders, further emphasizing the key role of the gut barrier in variceal bleeding and its sequelae. FABP2, an endogenous cytosolic enterocyte protein, is a marker of enterocyte integrity. Because FABP2 is located in the mature epithelium of the villi, it can more easily leak into the bloodstream when enterocytes are damaged. Animal and clinical studies have shown that cirrhosis is associated with gut barrier disruption (33), and increased intestinal permeability appears to be involved in the pathogenesis of cirrhosis-related complications. We have previously reported (34) that the expression of claudin 1 and occludin is decreased in the intestinal epithelial cells of cirrhotic patients, especially in decompensated cirrhosis, indicating gut barrier impairment. Similarly, another study found increased duodenal permeability in patients with decompensated cirrhosis (35). In the same context, in the present study, we found that decreased TGF-β levels were associated with an increased risk of bleeding. TGF-β is an anti-inflammatory cytokine that protects the intestinal mucosal barrier (36); therefore, decreased TGF-β levels could potentially contribute to a disrupted gut barrier. Figure 4 shows our proposed mechanism for the contribution of bacterial translocation and increased gut permeability to variceal bleeding risk.

It is still unclear whether antibiotics significantly reduce bacterial translocation. Recently, a randomized controlled trial showed that rifaximin had no significant effect on indices of gut permeability and bacterial translocation in patients with decompensated cirrhosis (37, 38). In contrast, a previous study had reported that norfloxacin decreased endotoxin levels and partially reversed the hyperdynamic circulatory state in liver cirrhosis (39). Similarly, lactulose, which accelerates intestinal transit and improves gut permeability, reduced bacterial translocation and intestinal bacterial overgrowth in animal models of liver cirrhosis (40). In addition to antibiotics and lactulose, other anti-endotoxin agents such as bactericidal/permeability-increasing protein (BPI), high-density lipoprotein (HDL), anti-endotoxin antibodies, and LPS antagonists should be investigated as potential treatment strategies to inhibit bacterial translocation and thus reduce the risk of variceal bleeding and bleeding-related mortality in cirrhotic patients. In addition, interventions to modulate the gut microbiota, including prebiotics, probiotics, and transplantation of fecal microbiota, may play a role in preventing bacterial translocation in chronic liver disease (41). Probiotics and prebiotics have been shown to be effective in reducing bacterial translocation in patients with diabetes mellitus (42), HIV (43), and in cancer patients after surgery (44). Horvath et al. (45) studied the effect of probiotic treatment in patients with liver cirrhosis and found minimal benefits in terms of gut barrier function and prevention of bacterial translocation. The critical role of bacterial translocation and gut barrier permeability in the development of variceal bleeding underscores the need for further randomized controlled trials investigating the role of microbiota-modulating treatments in the prevention of variceal bleeding and cirrhosis-related prognosis.

Regarding 6-week mortality, our analysis showed that it correlated with FABP2, albumin, CRP levels, severity of liver disease, and the presence of severe cirrhosis-related complications, including portal vein thrombosis and hepatocellular carcinoma. The presence of hepatocellular carcinoma has been shown to be an independent predictor of early rebleeding or mortality in patients with variceal bleeding (46–48). In a recent study, the presence and stage of hepatocellular carcinoma were identified as strong predictors of 6-week mortality in patients with acute variceal bleeding (49). In parallel, a new model for predicting prognosis in patients with cirrhosis and acute gastrointestinal bleeding, called the CAGIB score, has recently been developed (50). Among other clinical and laboratory variables, hepatocellular carcinoma is a component of this model (50). Considering the prognostic importance of hepatocellular carcinoma for the outcomes of patients with cirrhosis and variceal bleeding, we included patients with cirrhosis and hepatocellular carcinoma in our study. In addition, there was no difference in the presence of cirrhotics with hepatocellular carcinoma between the studied groups (*p*-value 0.137). Despite the widespread use of Child-Pugh and MELD scores as predictive models of cirrhosis progression, several investigators advocate the use of alternative laboratory indices, including CRP levels (3, 51, 52). In a recent study, Lee et al. (3) showed that CRP levels strongly predicted 6-week mortality after acute variceal bleeding. The authors suggested that the prognostic value of CRP should be explained by the presence of infection or altered inflammatory response, both conditions associated with elevated CRP levels.

A limitation of the present study was the relatively short follow-up period of survivors of variceal bleeding. A longer follow-up period and additional measurements of indices of gut permeability and bacterial translocation would likely have allowed us to draw more robust conclusions about the pathophysiology of variceal bleeding. Another limitation was the small sample size, which did not allow further analysis to determine significant predictors.

In conclusion, the present study provides direct evidence of the contribution of bacterial translocation and increased gut permeability to variceal bleeding risk. Our findings pave the way for future research to elucidate the role of microbiota in variceal bleeding and to investigate the therapeutic effects of microbiota-modulating interventions and anti-endotoxin agents on bleeding risk and survival of cirrhotic patients.

## Data Availability Statement

The raw data supporting the conclusions of this article will be made available by the authors, without undue reservation.

## Ethics Statement

The studies involving human participants were reviewed and approved by Patras University Hospital Scientific Review Board and Ethics Committee. The patients/participants provided their written informed consent to participate in this study.

## Author Contributions

CT had the idea, drafted the article, wrote the article, critically revised the article for important intellectual content, and approved publication of the article. MK, SA, and KK collected the data, analyzed and interpreted the data, wrote the article, and approved publication of the article. AMa, IA, PS, GT, MR, HK, and DG collected the data, analyzed and interpreted the data, and approved publication of the article. SM, CG, DS, GD, AMo, and KT critically revised the article for important intellectual content and approved publication of the article. All authors contributed to the article and approved the submitted version.

## Conflict of Interest

The authors declare that the research was conducted in the absence of any commercial or financial relationships that could be construed as a potential conflict of interest.

## Publisher's Note

All claims expressed in this article are solely those of the authors and do not necessarily represent those of their affiliated organizations, or those of the publisher, the editors and the reviewers. Any product that may be evaluated in this article, or claim that may be made by its manufacturer, is not guaranteed or endorsed by the publisher.
