# Supplementary material for: Endotoxin Translocation and Gut Barrier Dysfunction Are Related to Variceal Bleeding in Patients With Liver Cirrhosis
Source: Front Med (Lausanne). 2022 Mar 3;9:836306. doi: 10.3389/fmed.2022.836306 (PMC8929724; doi:10.3389/fmed.2022.836306)
Supplement: Supplementary file 1 [file Data_Sheet_1.docx]

Supplementary Material

**Supplementary Table 1.** Laboratory parameters of patients with stable liver cirrhosis and patients presenting with variceal bleeding

|  | **All study subjects** | **Variceal bleeders** | **Stable cirrhotics** | **p value** |
| --- | --- | --- | --- | --- |
|  | **Median (IQR)** | | |  |
| **IgM (MMU/ml)** | 38.8 (26.4, 79.7) | 36.2 (20.9, 64.3) | 52.8 (30.8, 112.8) | **0.034** |
| **IgG (GMU/ml)** | 237.8 (163.0, 352.2) | 229.8 (169.0, 360.1) | 261.7 (143.8, 371.4) | 0.881 |
| **FABP2 (pg/ml)** | 1868.4 (1256.7, 3573.2) | 2247.2 (1524.2, 4792.2) | 1468.7 (815.6, 1900.8) | **0.042** |
| **CD14 (pg/mL)** | 2499.1 (1657.6, 3526.9) | 2945.3 (1854.4, 3589.5) | 2338 (1553.1, 3361) | 0.506 |
| **TGF-β (pg/ml)** | 4 (2.8, 5.6) | 3.3 (2.4, 5.0) | 4.8 (3.3, 6.1) | **0.002** |
| **IL-1β (pg/ml)** | 9.4 (0, 12.1) | 9.4 (0, 11.6) | 9.4 (0, 13.4) | 0.824 |
| **IL-6 (pg/ml)** | 15.8 (8.5, 34.4) | 23.1 (14.7, 47.4) | 11 (5.5, 21.7) | **0.001** |
| **IL-8 (pg/ml)** | 131.8 (59.6, 282.4) | 142.3 (72.5, 304.1) | 131.5 (30.5, 275.1) | 0.231 |
| **IL-12 (pg/ml)** | 9.9 (0, 15.8) | 10.4 (0, 15.5) | 4.6 (0, 16.1) | 0.340 |
| **IL-10 (pg/ml)** | 5 (0, 8.5) | 5.9 (0, 8.5) | 0 (0, 8.4) | 0.519 |
| **TNF-α (pg/ml)** | 5.8 (0, 8.6) | 6.4 (0, 10.1) | 5.2 (0, 7.9) | 0.241 |
| **Endotoxin (EU/mL)** | 3.3 (1.7, 11) | 3.4 (1.9, 17.6) | 2.6 (1.6, 4.8) | 0.497 |
| **LBP (μg/mL)** | 5.0 (3.8-8.3) | 5.7 (3.4-10.1) | 4.5 (3.8, 5.7) | 0.351 |
| **NO (μM)** | 7.6 (3.8-14.0) | 5.0 (2.8-11.0) | 9.2 (5.5, 14.7) | **0.017** |

IQR, interquartile range; FABP2, fatty acid-binding protein 2; LBP, lipoprotein binding protein; NO, nitrogen oxide.

**Supplementary Table 2.** Laboratory parameters of patients with uncontrolled bleeding vs patients with controlled bleeding

| **Variceal bleeders** | **Failure to control bleeding** | **Controlled bleeding** | **p-value** |
| --- | --- | --- | --- |
|  | **Median (IQR)** | |  |
| **IgM (MMU/ml)** | 37.2 (12.3, 62.1) | 36.2 (21.1, 66.1) | 0.511 |
| **IgG (GMU/ml)** | 261.15 (156.2, 366,1) | 229.8 (174.9, 341.2) | >0.999 |
| **FABP2 (pg/ml)** | 2123.65 (2116.4, 2130,9) | 2569 (1437.55, 4508.70) | 0.882 |
| **CD14 (pg/mL)** | 1155.5 (973.5, 1337.5) | 3232.7 (1961.3, 3708.6) | **0.006** |
| **TGF-β (pg/ml)** | 4.74 (2.35, 7.32) | 3.27 (2.36, 4.55) | 0.406 |
| **IL-1β (pg/ml)** | 10.16 (2.32, 11.85) | 9.21 (0.00, 11.56) | 0.778 |
| **IL-6 (pg/ml)** | 33.65 (24.36, 45.60) | 18.06 (13.89, 60.05) | 0.365 |
| **IL-8 (pg/ml)** | 136.85 (74.50, 263.83) | 142.27 (72.46, 350.56) | 0.782 |
| **IL-12 (pg/ml)** | 7.85 (0.00, 16.13) | 10.42 (0.00, 14.47) | 0.837 |
| **IL-10 (pg/ml)** | 3.05 (0.00, 6.73) | 6.12 (0.00, 9.09) | 0.347 |
| **TNF-α (pg/ml)** | 3.05 (0.00-7.38) | 6.55 (1.29, 10.54) | 0.285 |
| **Endotoxin (EU/mL)** | 20.38 (6.71, 34.37) | 3.38 (1.81, 16.27) | 0.087 |
| **LBP (μg/mL)** | 3.59 (0.80, 5.17) | 5.96 (4.31, 10.23) | 0.098 |
| **NO (μM)** | 11.84 (7.49, 20.72) | 4.20 (2.48, 8.74) | **0.050** |

IQR, interquartile range; FABP2, fatty acid-binding protein 2; LBP, lipoprotein binding protein; NO, nitrogen oxide.

**Supplementary Table 3.** Laboratory parameters in 6-week survivors vs non-survivors

| **Variceal bleeders** | **6-week survivors** | **Non-survivors** | **p value** |
| --- | --- | --- | --- |
|  | **Median (IQR)** | |  |
| **IgM (MMU/ml)** | 30.4 (20.8, 72.5) | 40.35 (31.8, 64.33) | 0.626 |
| **IgG (GMU/ml)** | 224.5 (174.9, 341.2) | 274.95 (144.88, 510.63) | 0.670 |
| **FABP2 (pg/ml)** | 2058.6 (1324.5, 3184.2) | 4298.9 (2127.28, 9011.53) | **0.025** |
| **CD14 (pg/mL)** | 3232.7 (1961.3, 3529.0) | 1802.1 (1140.98, 4121.2) | 0.361 |
| **TGF-β (pg/mL)** | 3.2 (2.4, 4.9) | 3.61 (2.1, 6.37) | 0.907 |
| **IL-1β (pg/mL)** | 9.9 (0, 11.9) | 9.26 (0, 11.05) | 0.654 |
| **IL-6 (pg/mL)** | 18.0 (14.2, 43.9) | 42.18 (24.1, 67.76) | 0.114 |
| **IL-8 (pg/mL)** | 119.7 (65.6, 253.5) | 299.9 (155.6, 372.32) | 0.069 |
| **IL-12 (pg/mL)** | 11.6 (4.2, 15.6) | 0 (0, 15.69) | 0.122 |
| **IL-10 (pg/mL)** | 6.9 (0, 9.0) | 0 (0, 6.1) | 0.309 |
| **TNF-α (pg/mL)** | 6.6 (5.2, 10.4) | 0 (0, 10.1) | 0.182 |
| **Endotoxin (EU/mL)** | 3.3 (1.8, 12.4) | 17.47 (2.19, 20.28) | 0.202 |
| **LBP (μg/mL)** | 6.0 (2.7, 10.4) | 5.19 (2.89, 6.28) | 0.403 |
| **NO (μM)** | 4.8 (2.8, 9.3) | 7.4 (2.24, 19.12) | 0.603 |

IQR, interquartile range; FABP2, fatty acid-binding protein 2; LBP, lipoprotein binding protein; NO, nitrogen oxide.
